# Treatment of advanced soft-tissue sarcomas using a combined strategy of high-dose ifosfamide, high-dose doxorubicin and salvage therapies

**DOI:** 10.1038/sj.bjc.6603420

**Published:** 2006-10-10

**Authors:** S Leyvraz, R Herrmann, L Guillou, H P Honegger, A Christinat, M F Fey, C Sessa, M Wernli, T Cerny, D Dietrich, B Pestalozzi

**Affiliations:** 1 Centre Pluridisciplinaire d'Oncologie, University Hospital, CHUV BH06, Rue du Bugnon 46, 1011 Lausanne, Switzerland; 2 Medical Oncology Clinic, University Hospital, Basel, Switzerland; 3 Pathology Institute, University Hospital, Lausanne, Switzerland; 4 Triemli City Hospital, Zurich, Switzerland; 5 Institute of Medical Oncology, Inselspital, Bern, Switzerland; 6 Istituto Oncologico della Svizzera italiana, Bellinzona, Switzerland; 7 Oncology Hematology Department, Kantonsspital, Aarau, Switzerland; 8 Oncology/Hematology, Kantonsspital, St. Gallen, Switzerland; 9 SAKK Coordinating Center, Bern, Switzerland; 10 Department of Oncology, University Hospital, Zurich, Switzerland

**Keywords:** doxorubicin, ifosfamide, salvage surgery, soft-tissue sarcomas

## Abstract

Having determined in a phase I study the maximum tolerated dose of high-dose ifosfamide combined with high-dose doxorubicin, we now report the long-term results of a phase II trial in advanced soft-tissue sarcomas. Forty-six patients with locally advanced or metastatic soft-tissue sarcomas were included, with age <60 years and all except one in good performance status (0 or 1). The chemotherapy treatment consisted of ifosfamide 10 g m^−2^ (continuous infusion for 5 days), doxorubicin 30 mg m^−2^ day^−1^ × 3 (total dose 90 mg m^−2^), mesna and granulocyte-colony stimulating factor. Cycles were repeated every 21 days. A median of 4 (1–6) cycles per patient was administered. Twenty-two patients responded to therapy, including three complete responders and 19 partial responders for an overall response rate of 48% (95% CI: 33–63%). The response rate was not different between localised and metastatic diseases or between histological types, but was higher in grade 3 tumours. Median overall survival was 19 months. Salvage therapies (surgery and/or radiotherapy) were performed in 43% of patients and found to be the most significant predictor for favourable survival (exploratory multivariate analysis). Haematological toxicity was severe, including grade ⩾3 neutropenia in 59%, thrombopenia in 39% and anaemia in 27% of cycles. Three patients experienced grade 3 neurotoxicity and one patient died of septic shock. This high-dose regimen is toxic but nonetheless feasible in multicentre settings in non elderly patients with good performance status. A high response rate was obtained. Prolonged survival was mainly a function of salvage therapies.

Soft-tissue sarcomas are a highly heterogeneous group of tumours with a low incidence. At least 50 different subtypes are distinguished, each with its specific biology and clinical outcome. The majority of these tumours are presumably derived from mesoderm and categorised by the normal tissue they resemble. As they are uncommon, however, they are subsumed under the collective term *soft-tissue sarcoma* and therapeutically approached in similar ways.

In recent years, progress has been made in understanding their clinical and biological complexity (Dirix and Van Oosterom, 1999; Fayette and Blay, 2005), such that oncologists have increasingly been able to define customised therapies. Paclitaxel has been found to be active mainly in patients with angiosarcomas (Fata *et al*, 1999). Docetaxel combined with gemcitabine has been shown to induce a 53% response rate in leiomyosarcomas of gynaecological origin (Hensley *et al*, 2002). Topoisomerase inhibitors are active for rhabdomyosarcomas (Cosetti *et al*, 2002). Trabectedin (ET743) has a low objective response rate but led to stable disease in 24% of pretreated patients (Yovine *et al*, 2004) and was mainly active in myxoid liposarcomas (Grosso *et al*, 2006).

The most common approach used in all histological subtypes, however, is to use doxorubicin and ifosfamide (Edmonson *et al*, 1993). Their anti-tumour activity as single agents does not exceed 15–25%. Combining these agents might be more effective but has not been shown to improve survival in a significant way compared to their use as single agents (Santoro *et al*, 1995).

Following suggestions that these agents may have a dose–response relationship (O'Bryan *et al*, 1973; Le Cesne *et al*, 1995; Cerny *et al*, 1999), various phase-I and -II trials were performed in which they were administered at high doses, yielding response rates of 40–66% (Frustaci *et al*, 1997; Patel *et al*, 1998; Reichardt *et al*, 1998; Maurel *et al*, 2004a). In some studies, median overall survival was up to 24 months. There are also suggestions that applying regimens with high response rates may render an initially inoperable tumour operable, thereby improving the outcome (Judson, 2004).

In a previous study, we defined the maximum tolerated dose of doxorubicin combined with ifosfamide, administered every 3 weeks and supported by haematopoietic growth factors (Leyvraz *et al*, 1998). The maximum tolerated dose of doxorubicin was 60 mg m^−2^ with ifosfamide at 12 g m^−2^ and could be increased to 90 mg m^−2^ with ifosfamide at 10 g m^−2^. Myelosuppression was severe but resolved within 4 days.

On this basis, we designed a multicentre nonrandomised phase-II trial for advanced and metastatic soft-tissue sarcomas. Salvage surgery and/or radiotherapy with the aim of removing all residual tumour tissue was performed whenever such an approach was considered appropriate to render patients free of disease.

## PATIENTS AND METHODS

The protocol was in accordance with the Declaration of Helsinki principles and was approved by the respective responsible ethics commissions. All patients included in the study gave their written informed consent.

### Inclusion criteria

Patients with histologically demonstrated advanced or metastatic soft-tissue sarcoma were eligible for the study. Excluded were patients with histological evidence of malignant mesothelioma, chondrosarcoma, neuroblastoma, osteosarcoma, Ewing sarcoma and embryonal rhadomyosarcoma.

Additional eligibility criteria included the presence of measurable disease in one or two dimensions as demonstrated by physical examination or imaging techniques. Ascites and pleural effusions were not considered measurable. Patients were only included if they were 18–70 years old and showed a performance status of ⩽2 as defined by the Eastern Cooperative Oncology Group (ECOG) (Oken *et al*, 1982). They were not included in the presence of previous chemotherapy, previous radiotherapy on recurrent disease, or CNS metastases. Another exclusion criterion was abnormal haematological, renal or hepatic function. Cardiac function was required to be within the normal range on echocardiography or multigated nuclear scanning.

### Study design and treatment plan

This was a multicentre, single-arm phase II trial (protocol SAKK 57/93). Chemotherapy cycles were repeated every 3 weeks as follows: (i) ifosfamide 10 g m^−2^ i.v. as continuous infusion over 5 days; (ii) mesna 2 g m^−2^ day^−1^ i.v. as continuous infusion over 6 days; (iii) doxorubicin 30 mg m^−2^ day^−1^ i.v. bolus given for 3 days (total dose 90 mg m^−2^), starting 4 h after initiation of ifosfamide on day 1; and (iv) granulocyte-colony stimulating factor (G-CSF, Filgrastim) 5 *μ*g kg^−1^ s.c. once daily as of day 6 for 10 days or – if neutrophile counts were <500 *μ*l^−1^ by that time – for a total of 14 days.

## RESPONSE, TOXICITY ASSESSMENT, DOSE MODIFICATION AND PATHOLOGY REVIEW

Response in up to three measurable lesions was assessed by tumour evaluation according to standard WHO criteria (World Health Organization, 1979). Each observed complete or partial response had to be confirmed at least 4 weeks later. CT scans were obtained at baseline, after every other chemotherapy cycle, and at the end of the treatment. Toxicity was assessed based on WHO grading, and neurotoxicity based on MD Anderson scores (Castellanos and Fields, 1986).

Subsequent treatment cycles were delayed by 1 or 2 weeks if <3500 *μ*l^−1^ leukocytes or <100 000 *μ*l^−1^ platelets were measured at day 22. Dose modifications were performed: (i) if i.v. antibiotics were required during myelosuppression or in case of thrombocytopenia-related bleeding, by reducing ifosfamide to 8 g m^−2^; (ii) in the presence of WHO grade ⩾3 mucositis, by reducing doxorubicin to 75 mg m^−2^. The dose of doxorubicin was not reduced after myelosuppression. Ifosfamide could be increased to 12 g m^−2^ after normal recovery from myelosuppression without febrile episodes requiring the use of i.v. antibiotics. Dose intensity was calculated in mg m^−2^ week^−1^ for each patient (Hryniuk and Goodyear, 1990).

In case the MD Anderson score was ⩾2, an attempt was made to manage the neurotoxic event by administering methylene blue rather than decreasing the dose of ifosfamide. Methylene blue was then administered as an i.v. bolus of 50 mg repeated at the same dose every 2 h, or as continuous infusion at 200 mg day^−1^ diluted in 5% dextrose, until the event resolved (Küpfer *et al*, 1994).

Cardiotoxicity was monitored by assessing cardiac ejection fraction every 2–3 cycles and prior to chemotherapy cycles on reaching doxorubicin cumulative doses of >450 mg m^−2^. Chemotherapy was discontinued if cardiac ejection fraction dropped to <40%. Chemotherapy was not administered in the presence of serum creatinine >150 *μ*mol l^−1^ or creatinine clearance <60 ml min^−1^.

Pathology specimens were reviewed by an independent group of expert pathologists led by LG to confirm diagnosis and tumour grade based on the FNCLCC grading system (Guillou *et al*, 1997).

### Statistical methods

The primary end point of the study was the tumour response rate based on WHO criteria. Simon's optimal two-stage design was used to calculate the number of patients to be included in the phase-II trial, based on the premise that a response rate of <40% would disqualify the treatment regimen, while a response rate of ⩾70% would render it a promising option. To obtain a significance level of 5% at a power of 80%, a total number of 46 patients were required, with 16 of them in the stage-I and another 30 in the stage-II phase. After the first 16 patients had been evaluated for their response to chemotherapy, it was decided that the trial should be continued until the target number of 46 patients was reached. At least 24 responses among the 46 patients were required to consider the treatment promising.

Confidence intervals for response rates were calculated using the Clopper–Pearson method. Time to progression was calculated for all patients from the time of enrollment until disease progression or death, with censoring at last follow-up. Survival times were calculated from enrollment to death or last follow-up. The Kaplan–Meier was used to estimate the median values of time to progression and survival. Log-rank test was used to evaluate differences in survival. Cox regression was used for exploratory multivariate analysis of survival. The following variables, represented by appropriate binary indicators, were included in the Cox regression: histology (leiomyosarcomas *vs* other), tumour grade (3 *vs* other), response (complete/partial response *vs* other), tumour location (localised vs metastatic disease) and salvage therapy with curative intention (yes *vs* no).

## RESULTS

### Patient characteristics

Fifty-one patients with advanced soft-tissue sarcomas, from seven SAKK (Swiss Group for Clinical Cancer Research) centres were enrolled in the study between 10/1995 and 1/2001. As gastro-intestinal stromal tumour (GIST) is now recognised as a specific entity, it was decided to exclude patients with GIST from analysis. On histopathological review, five patients were excluded from analysis because of the following characteristics: melanoma, progressive disease after one cycle of the studied regimen (*n*=1); cystosarcoma phyllodes of the breast with metastases in the lung, partial response after six cycles of treatment (*n*=1); three GIST, progression after two chemotherapy cycles (*n*=2) and death due to intestinal haemorrhage during the first chemotherapy cycle (*n*=1).

Thus, a total of 46 patients (18 women and 28 men) could be evaluated. Pertinent characteristics are summarised in Table 1. The median age of these patients was 51 (21–60) years. Except one patient had a compromised performance status WHO grade 2, all the others had a WHO grade 0 or 1. Leiomyosarcomas and malignant fibrohistiocytoma (MFH) were the most frequent histologies, each accounting for almost one-third of all tumour types. Tumours were grade 3 in 54% and grade 2 in 30% of patients. The six patients with grade 1 tumour (13%) diagnosed on the biopsy of the primary had distant metastases at time of inclusion. While most patients were diagnosed with metastatic tumours, one-third presented with advanced localised disease (large peripheral primary=5, inoperable primary=4, recurrences=5).

### Toxicity and dose intensity

A median of 4 (1–6) chemotherapy cycles per patient was administered, and a total of 187 cycles were analysed for toxicity. Myelosuppression was the predominant type of toxicity. Severe neutropenia WHO ⩾grade 3 was observed in 59% of cycles (73% of patients being affected) and a febrile episode occurred in 29% (with demonstrable infection in 20%) of cycles. One patient died of septic shock during his fourth cycle. WHO ⩾grade 3 thrombocytopenia and anaemia were observed in 39 and 27% of cycles, respectively, affecting two-third of patients.

Neurological toxicity (MD Anderson score) was observed during 11 cycles (grade 1: *n*=8; grade 3: *n*=3). All events could be managed by administration of methylene blue and/or discontinuation of ifosfamide. Transient elevations in creatinine levels were observed in three patients. No cases of severe renal toxicity were noted, although 17 cycles were associated with transient microscopic haematuria. Mucositis occurred in 46% of cycles but was generally moderate, reaching grade 3 or 4 in only 19 cycles (10%). No cases of clinical heart failure were noted, although two patients had significant drops in cardiac ejection fraction from baseline. Doses were modified in 29% of cycles, invariably because of toxicity. A total of 22% chemotherapy cycles were delayed because of toxicity or upon patients' request. The dose intensity of ifosfamide could be maintained at a median of 2740 mg m^−2^ week^−1^ (82% of the planned dose) and for doxorubicin at a median of 26 mg m^−2^ week^−1^ (86% of the planned dose).

### Response and survival

Of the 46 patients who were evaluated for response to chemotherapy, three were complete responders (6.5%) and 19 were partial responders (41%). The overall response rate was thus 48% (98% CI: 33–63%). A total of 18 patients showed stable disease (39%), and five patients showed progressive disease (11%). The response rate was not different between localised and metastatic diseases (50 and 47%). A partial response was observed in two among the five patients with large primaries. Broken up by histological tumour types, overall response to chemotherapy was observed in 5/13 patients with leiomyosarcomas (38%, 95% CI: 14–68%), in 6/11 patients with malignant fibrohistiocytomas (54%, 95% CI: 23–83%), in 4/4 patients with epithelioid sarcomas, in 3/4 patients with angiosarcomas, in 1/4 patients with neurofibrosarcomas and in 0/4 patients with liposarcomas. Response rates were slightly higher in grade 3 (14/25=56%, 95% CI: 35–76%) than in grade 1–2 (8/20=40%, 95% CI: 19–64%) tumours.

A total of 20 patients were treated by salvage surgery and/or radiotherapy following partial response (*n*=12) or stable disease (*n*=8) after chemotherapy. These salvage regimens included both surgery and radiotherapy (*n*=7), surgery (*n*=2) or radiotherapy (*n*=1) in 10 patients with localised disease and surgery (*n*=6) or radiotherapy (*n*=4) in 10 patients with metastatic disease.

**Figure 1:**
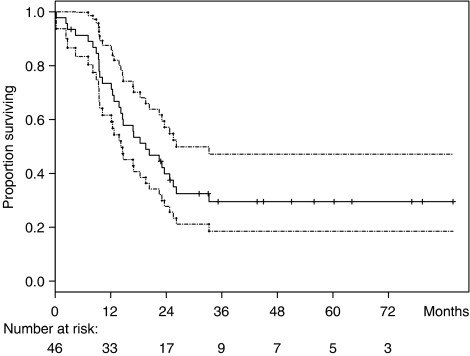
Kaplan–Meier curve for overall survival (interrupted line 95% confidence interval).

**Figure 2:**
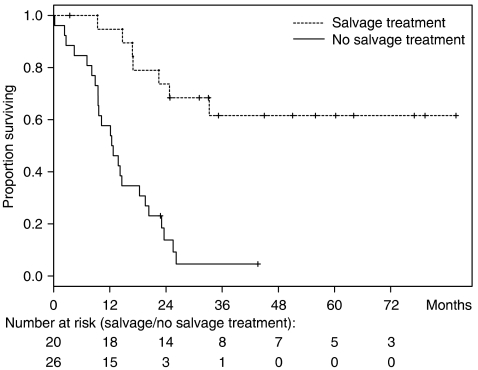
Overall survival of patients treated by salvage surgery and/or radiotherapy (aiming to eliminate all residual disease) in addition to chemotherapy *vs* patients treated by high-dose chemotherapy only.

After a median follow-up of 45 months, there were 31 cases of tumour progression and 24 cases of tumour-related death. Other causes of death included coronary artery disease (*n*=1), pulmonary embolism (*n*=2), septic shock (*n*=1) or remained unknown (*n*=3). Overall, our strategy yielded a median time to progression of 16.2 months (95% CI: 10.2–22.5) and a median overall survival of 19.6 months (95% CI: 14.2–25.5) (Figure 1). Multivariate analysis including salvage therapy, histology, grade, tumour localisation and response to chemotherapy as covariates demonstrated that salvage therapy was the most significant predictor for favourable outcomes (hazard ratio: 0.11; 95% CI: 0.04–0.30). Leiomyosarcomas were associated with significantly better outcomes than all other histological tumour types (hazard ratio: 0.27; 95% CI: 0.11–0.70). By contrast, tumour grade, localisation and tumour response to chemotherapy had any significant impact on survival (Figure 2).

## DISCUSSION

In a previous phase I trial, we identified the maximum tolerated dose levels for combined administration of ifosfamide and doxorubicin (Leyvraz *et al*, 1998). The phase II trial herein presented was required to confirm the increased response rate suggested in that previous trial. It has successfully accomplished this purpose by demonstrating a response rate of 48% (95% CI: 33–63%) in 46 patients with advanced soft-tissue sarcomas. Although this observed response rate did not reach the requirement being considered promising as defined by the statistical design, it is in the range of similar response rates (40–66%) for high-dose combination of ifosfamide and doxorubicin that have been obtained in other phase-II studies (Frustaci *et al*, 1997; Patel *et al*, 1998; Reichardt *et al*, 1998). The most recent trials have yielded less impressive response rates (25–23%) but were designed such that only one agent was administered at high dose levels (Le Cesne *et al*, 2000; Worden *et al*, 2005). Indeed, the efficacy of ifosfamide alone at standard dose (5 g m^−2^) was shown to be minimal (10%) in a randomised trial (van Oosterom *et al*, 2002). Moreover the best schedule of ifosfamide administration is still unknown. There were suggestions that daily 2- to 4 h-infusion might be more active than continuous infusion (Patel *et al*, 1997), but might be also more toxic (Antman *et al*, 1989).

As expected, and despite the use of haematopoietic growth factors, the combined myelotoxicity of both agents employed in our study led to severe (WHO grade 3–4) neutropenia in 59% of cycles and 73% of patients. These episodes were of short duration. Febrile neutropenia occurred in 29% of cycles. One patient died of septic shock. Severe anaemia and thrombopenia were observed in two-thirds of patients. Based on our results, we conclude that these toxic events can be managed in a multicentre setting if adequate experience is available in the participating centres, but limited to patients aged ⩽60 years and with a performance status <2.

Similar toxicity profiles, with severe neutropenia in 94–100% of patients, were described in other trials (Patel *et al*, 1998; Reichardt *et al*, 1998). Lower-dose combinations led to severe neutropenia in 87–90% and severe infection in 17% of patients (Le Cesne *et al*, 2000; Worden *et al*, 2005). One of these studies involved two toxic deaths out of 40 patients, severe thrombopenia in 50–62% and severe anaemia in approximately 50% of patients (Worden *et al*, 2005). Even standard dose levels (ifosfamide 6 g m^−2^ and doxorubicin 60 mg m^−2^ and G-CSF) led to severe neutropenia in 49%, anaemia in 23%, and thrombopenia in 15% of patients (Worden *et al*, 2005). In a large-scale EORTC trial, dose levels of ifosfamide 5 g m^−2^ and doxorubicin 50 mg m^−2^ but without G-CSF led also to severe neutropenia in 86%, and thrombopenia in 8% of patients (Le Cesne *et al*, 2000).

It is thus reasonable to conclude that myelosuppression should be accepted as inherent in any ifosfamide-doxorubicin combination therapy. Therefore, these regimens should only be administered in specialised centres (Benjamin, 1987). If severe toxicity is the price to be paid for improved outcomes, it might be acceptable after all.

Median overall survival in the present study was 19.6 months, with an estimated 5-year-survival rate of 30%. These results are comparable with overall survival times of 18–25 months reported in other intensive-dose trials (Frustaci *et al*, 1997; De Pas *et al*, 1998; Reichardt *et al*, 1998). They are presumably also better than the 12 months obtained in standard-dose trials, even though the respective data may not be suitable for direct comparisons (Bramwell *et al*, 2003). The exploratory multivariate analysis defined that leiomyosarcoma had a better outcome compared to other histology subtypes. The prognostic value on survival of the different histologies is yet unsettled. When standard chemotherapy is used, the median overall survival was improved for liposarcoma (Van Glabbeke *et al*, 1999), but 5-year survivors could be found equally in all subtypes (Blay *et al*, 2003). If leiomyosarcomas have a very low response rate (10%) with standard chemotherapy, in our small series, as well as in other similar ones, the response rates ranged between 30 and 75% (Frustaci *et al*, 1997; Reichardt *et al*, 1998; Maurel *et al*, 2004b), potentially explaining an improved outcome.

The objection may be raised that our results were only possible because we selected patients with favourable prognostic factors and good performance status who were in a particularly good condition to tolerate such an intensive and toxic approach (Van Glabbeke *et al*, 1999). While this may be true, the EORTC has demonstrated (based on 4% of 1888 patients) that complete remission is not just a yardstick for chemotherapeutic success but is really the most important predictor of favourable long-term survival (Blay *et al*, 2003).

Trials in which high-dose ifosfamide was combined with high-dose anthracylines have yielded complete remission rates of 8–20% (Reichardt *et al*, 1998). In our study it was 6.5% and might have accounted only marginally to the final results. However, a complete remission should not be solely based on chemotherapy. Salvage surgery performed after chemotherapy should also be taken into account, since patients in whom all residual disease is surgically removed will do equally well (Reichardt *et al*, 1998). In 117 patients that have been operated in order to render them without evidence of disease, a median overall survival of 19 months was obtained (Billingsley *et al*, 1999). In previous studies of high-dose chemotherapy, salvage surgery with or without radiotherapy could be performed in 17–32% of patients and may have contributed to the improved outcomes in these series.

With these considerations in mind, high-dose chemotherapy with G-CSF support could be defined as part of a multidisciplinary strategy in the treatment of soft-tissue sarcomas, to be performed at experienced institutions only. Its goal would be to induce the most effective antitumour response available, thereby preparing the ground for salvage therapies to improve the outcome further. And future research should focus on identifying patients that might benefit form such a combined treatment approach.

## Figures and Tables

**Table 1:** Patient characteristics

**Number of patients**	**46**
*Sex*	
Male	28
Female	18
	
*Performance status*	
0	24
1	21
2	1
	
*Sarcoma type*	
Leiomyosarcoma	13
Malignant fibrous histiocytoma	11
Angiosarcoma	4
Epithelioid sarcoma	4
Liposarcoma	4
Neurofibrosarcoma	4
Unclassified	2
Alveolar rhabdomyosarcoma	1
Synoviosarcoma	1
Extra-skeletal osteosarcoma	1
Malignant hemangiopericytoma	1
	
*Differentiation grade*	
1	6
2	14
3	25
Unknown	1
	
*Tumour location*	
Localised	14
Metastatic	32
Lung	21
Lymph nodes	13
Liver	6
Bone	3
Soft tissue	3
Pancreas	1
Kidney	1
Peritoneum	1
